# Impact of Graft Weight Change During Perfusion on Hepatocellular Carcinoma Recurrence After Living Donor Liver Transplantation

**DOI:** 10.3389/fonc.2020.609844

**Published:** 2021-02-24

**Authors:** Jong Man Kim, Young Jae Chung, Sangjin Kim, Jinsoo Rhu, Gyu-Seong Choi, Jae-Won Joh

**Affiliations:** Department of Surgery, Samsung Medical Center, Sungkyunkwan University School of Medicine, Seoul, South Korea

**Keywords:** living donors, partial liver graft, perfusion, outcomes, hepatocellular carcinoma

## Abstract

**Backgrounds:**

Inadequate liver volume and weight is a major source of morbidity and mortality after adult living donor liver transplantation (LDLT). The purpose of our study was to investigate HCC recurrence, graft failure, and patient survival according to change in right liver graft weight after histidine-tryptophan-ketoglutarate (HTK) solution perfusion in LDLT.

**Methods:**

Two hundred twenty-eight patients underwent LDLT between 2013 and 2017. We calculated the change in graft weight by subtracting pre-perfusion graft weight from post-perfusion graft weight. Patients with increased graft weight were defined as the positive group, and patients with decreased graft weight were defined as the negative group.

**Results:**

After excluding patients who did not meet study criteria, 148 patients underwent right or extended right hepatectomy. The negative group included 89 patients (60.1%), and the positive group included 59 patients (39.9%). Median graft weight change was -28 g (range; -132–0 g) in the negative group and 21 g (range; 1–63 g) in the positive group (P<0.001). Median hospitalization time was longer for the positive group than the negative group (27 days vs. 23 days; P=0.048). There were no statistical differences in tumor characteristics, postoperative complications, early allograft dysfunction, or acute rejection between the two groups. Disease-free survival, death-censored graft survival, and patient survival were lower in the positive group than the negative group. Additionally, the positive group showed strong association with HCC recurrence, death-censored graft survival, and patient survival in multivariate analysis.

**Conclusion:**

This study suggests that positive graft weight change during HTK solution perfusion indicates poor prognosis in LDLT.

## Introduction

In Asia, where there is a shortage of deceased donors, living donor liver transplantation (LDLT) is frequently performed in hepatocellular carcinoma (HCC) patients using right or left hemi-liver graft from living donors. Grafts from living donors are higher quality than livers from deceased donors. Numerous factors have been identified as predictors of short-term outcomes of LDLT such as age, model for end-stage liver disease (MELD) score, cold ischemic time, graft-to-recipient weight ratio (GRWR), transfusion demands, and laboratory test findings (1). Among these, graft volume and weight directly affect recipient recovery and graft survival because the volume of the implanted partial liver graft is less than the metabolic demand (2).

Histidine-Tryptophan-Ketoglutarate (HTK) solution is the only organ preservation solution used in Korea (3). HTK solution has a low viscosity index and low potassium and sodium levels (4). The low viscosity in HTK solution favors efficient liver graft washout because it flushes rapidly, and diffusion of preservation solution to the hepatocytes arrests hepatocellular damage. Additionally, HTK solution contains histidine acting as a buffer, tryptophan as a membrane stabilizer, and ketoglutarate acting as a substrate during ischemia (4). Because HTK solution is safe and effective, ICU stay, primary dysfunction rate, and biliary complication after liver transplantation are lower when HTK solution is used than when other preservation solutions are used (4).

Partial liver grafts are flushed with a chilled HTK solution through the portal vein in the back-table procedure. After this process, the liver graft weight decreases after perfusion compared to before perfusion. However, some patients have increased graft weight after perfusion. No previous study has investigated the effect of right liver graft weight change during organ preservation solution perfusion on recipient outcomes in LDLT patients. Herein, we investigated HCC recurrence, graft failure, and patient survival according to right liver graft weight change in adult LDLT patients.

## Patients and Methods

### Study Population

Two hundred twenty-eight patients underwent LDLT between January 2013 and March 2017 at Samsung Medical Center, Korea. There were 155 HCC patients who received LDLT with HCC. The present study was approved by the Institutional Review Board (IRB) of Samsung Medical Center (SMC-2020-07-027). The need for patient consent was waived by the IRB because this was a retrospective observational study of data that used patient medical records. We reviewed all patients’ medical records and excluded those who did not have records of graft weight (n=4), patients without HCC (n=48), multiple organ transplantation (n=2), re-transplantation (n=3), received a left-side graft (n=10), pediatric liver transplantation patients (n=8), or were lost during follow-up or had incomplete medical records (n=5). Finally, 148 HCC patients were identified in the study and underwent right or extended right hepatectomy.

### Immunosuppression Protocol

Our immunosuppressive regimen has been described in previous studies (5). Basiliximab was administered at a dose of 20 mg/day at the time of operation and on postoperative day 4. All patients received triple immunosuppressive drugs consisting of tacrolimus, mycophenolate mofetil (MMF), and methylprednisolone. Tacrolimus dose was adjusted to maintain whole-blood trough levels at 8–10 ng/ml for 1–2 months postoperatively and at 6–8 ng/ml thereafter. MMF was used at 500-1,000 mg/day postoperatively and adjusted according to white blood cell count. Methylprednisolone therapy was initiated on the operation day at a dose of 500 mg/day and then tapered to 4–8 mg/day within 1–2 months postoperatively. In case of ABO-incompatible LDLT, rituximab prophylaxis and frequent total plasma exchange were conducted for desensitization (6). When HCC recurs, tacrolimus trough level was lowered compared to patients without HCC recurrence and everolimus was added.

### Perfusion and Weight Measurements in Back-Table

At the beginning living liver donor surgery, liver wedge resection is performed in segment 4 to check for hepatic steatosis. The portal vein of the right liver graft is clamped using a vascular clamp, but the hepatic artery and hepatic vein are not clamped during liver graft extraction. The right liver graft comes out of the body immediately after right hepatic vein division and the vascular clamp is released. We manually and gently press the right liver graft to remove blood within the graft. The graft was then perfused with 3L of HTK solution *via* the portal vein.

We routinely measured the right liver graft weight twice before and after HTK preservation solution perfusion in the back-table procedure: (1) immediately after procurement after the blood was drained (blood-free graft weight) and (2) after perfusion with HTK solution (graft weight after perfusion). We calculated the change in liver graft weight by subtracting pre-perfusion graft weight from post-perfusion graft weight.

### Definitions

Patients with increased graft weight were defined as the positive group and patients with decreased graft weight as the negative group. GRWR was calculated by dividing graft weight by recipient body weight before and after perfusion. EAD was defined on the basis of abnormal increases in total bilirubin, international normalized ratio (INR), and aminotransferase within 7 days after LDLT (7).

### Statistical Analysis

To compare the differences between the positive and negative groups, Fisher’s exact test for categorical variables and the Mann-Whitney *U-*test for continuous variables were used. Continuous variables are expressed as median and range and categorical variables are expressed as number and percentage. Disease-free survival (DFS), death-censored graft survival, and patient survival (PS) were calculated as the duration from the starting date of LDLT to the date when a new event was first detected or, if the entire follow-up period was event-free, to the date of the last follow-up visit. DFS rates, death-censored graft survival rates, and PS rates between the positive and negative groups were estimated using the Kaplan-Meier method, and survival curves for those rates were compared with the log-rank test. Significant variables in univariate analyses (P < 0.05) were entered into a Cox multivariate proportional hazards model to determine which factors independently predicted DFS, death-censored graft survival, and patient survival.

All statistical analyses were performed using SPSS 24.0 for Windows (IBM Corp., Armonk, NY, USA). P < 0.05 was considered statistically significant and all statistical tests were evaluated as two-sided.

## Results

### Baseline Characteristics

There were 89 people (60.1%) in the negative group and 59 people (39.9%) in the positive group. Baseline characteristics are summarized in **Table 1**. Sex, age, and body mass index (BMI) in living liver donors were not significantly different between the two groups. Hepatitis B virus (HBV) was the most common cause of HCC in recipients, accounting for 79 cases (88.8%) in the negative group and 47 cases (79.7%) in the positive group. However, alcoholic damage (n=6, 10.2%) and HCV (n=5, 8.5%) were more common causes among the positive group than the negative group. There were no statistically significant differences in sex, age, body mass index, hypertension, diabetes, Child-Pugh class, MELD score, history of radiation, operation, radiofrequency ablation, and/or transarterial chemoembolization before LDLT, alpha-fetoprotein (AFP), and protein induced by vitamin K absence-II (PIVKA-II) between the two groups.

**Table 1:** Baseline characteristics in pre-transplant.

	Negative group (n=89)	Positive group (n=59)	P-value
**Donor**			
Sex (male)	56	33	0.476
Age (years)	28 (16–61)	31 (16–68)	0.183
Body mass index	23.1 (17.3–32.6)	22.8 (18.9–36.3)	0.771
**Recipient**			
Sex (male)	81 (91.0%)	53 (89.8%)	0.810
Age (years)	56 (43–70)	55 (37–68)	0.406
Body mass index	24.5 (17.3–35.6)	24.0 (18.3–36.7)	0.555
Hypertension	13 (14.6%)	4 (6.8%)	0.190
Diabetes	18 (20.2%)	9 (15.3%)	0.517
Diagnosis Alcoholic HBV HCV NBNC	2 (2.2%) 79 (88.8%) 2 (2.2%) 6 (6.7%)	6 (10.2%) 47 (79.7%) 5 (8.5%) 1 (1.7%)	0.026
Child-Pugh class A B C	46 (51.7%) 27 (30.3%) 16 (18.0%)	23 (39.0%) 20 (33.9%) 16 (27.1%)	0.127
MELD	10 (6–33)	11 (6–35)	0.266
History of radiation therapy in HCC	8 (9.0%)	5 (8.5%)	0.923
History of operation	15 (17.6%)	9 (16.1%)	0.808
History of RFA	33 (37.1%)	17 (28.8%)	0.375
History of TACE	64 (71.9%)	42 (71.2%)	0.924
AFP	7.2 (1.3–8,368)	5.0 (1.3–4,246)	0.093
PIVKA-II	27 (6–17,860)	26 (6–22,462)	0.315

*HBV, hepatitis B virus; HCV, hepatitis C virus; NBNC, non B non C; MELD, Model For End-Stage Liver Disease; HCC, hepatocellular carcinoma; RFA, radiofrequency ablation; TACE, transarterial chemoembolization; AFP, alpha-fetoprotein; PIVKA-II, proteins induced by vitamin K absence or antagonist-II.

### Perioperative and Pathologic Characteristics

Perioperative and pathologic characteristics are outlined in **Table 2**. Extended right hepatectomy was performed in two patients in the negative group and one patient in the positive group. All other patients underwent right hepatectomy. The incidence of laparoscopic liver resection in living liver donors was 33.7% (n=30) in the negative group and 28.8% (n=17) in the positive group. Median macrosteatosis and microsteatosis was 5% in both groups. The median donor operation time in both groups was also very similar (351 min in the negative group vs. 351 min in the positive group; P=0.893). For right lobe liver grafts extracted from a living donor, graft weight and GRWR before perfusion in the negative group were significantly greater than in the positive group (P=0.009 and P=0.020, respectively), but graft weight and GRWR after perfusion were no different between the two groups. Median graft weight change was -28 g (range; -132–0 g) in the negative group and 21 g (range; 1–63 g) in the positive group (P<0.001).

**Table 2:** Perioperative and postoperative characteristics.

	Negative group (n=89)	Positive group (n=59)	P-value
**Donor**			
Laparoscopic donor hepatectomy	30 (33.7%)	17 (28.8%)	0.591
Macrosteatosis (%)	5 (1–15)	5 (0–20)	0.176
Microsteatosis (%)	5 (1–70)	5 (1–40)	0.257
Donor operation time (min)	351 (187–632)	351 (189–581)	0.893
Graft weight at pre-perfusion (mg)	725 (524–1,170)	670 (520–990)	0.009
GRWR at pre-perfusion	1.06 (0.67–1.77)	1.00 (0.66–1.39)	0.020
Graft weight at post-perfusion (mg)	702 (502–1,169)	693 (534–1038)	0.956
GRWR at post-perfusion	1.00 (0.63–1.71)	1.00 (0.67–1.43)	0.897
Graft weight change (mg)	-28 (-132-0)	21 (1–63)	<0.001
**Recipient**			
ABO-incompatibility	20 (22.5%)	19 (32.2%)	0.253
Cold ischemic time (min)	87 (45–168)	90 (55–142)	0.409
Warm ischemic time (min)	36 (17–69)	38 (16–81)	0.221
Recipient operation time (min)	526 (336–960)	560 (351–838)	0.106
Early allograft dysfunction	2 (2.2%)	2 (3.4%)	0.675
Intensive care unit stay (days)	6 (2–11)	6 (2–17)	0.691
Hospitalization (days)	23 (17–197)	27 (20–445)	0.048
Milan criteria (Beyond)	30 (33.7%)	17 (28.8%)	0.587
Tumor size (cm)	2.4 (1.0–8.5)	2.5 (0.5–7.8)	0.398
Tumor number	2 (1–34)	2 (1–14)	0.666
Total tumor necrosis	37 (41.6%)	18 (30.5%)	0.217
Tumor grade 3 or 4	13 (14.6%)	6 (10.2%)	0.615
Encapsulation	64 (71.9%)	41 (69.5%)	0.844
Microvascular invasion	35 (39.3%)	22 (37.3%)	0.862
Portal vein thrombosis	5 (5.6%)	2 (3.4%)	0.703
Intrahepatic metastasis	23 (25.8%)	11 (18.4%)	0.421
Multicentric occurrence	21 (23.6%)	13 (22.0%)	0.839
**Postoperative characteristics in recipients**			
Bacterial infection within 90 days	7 (7.9%)	5 (8.5%)	0.894
Viral infection within 90 days	14 (15.7%)	10 (16.9%)	0.844
Surgical complications	33 (37.1%)	20 (33.9%)	0.863
Clavien-Dindo grade II IIIa IIIb	12 (13.5%) 11 (12.3%) 9 (10.1%)	8 (13.6%) 6 (10.2%) 6 (10.2%)	0.433
Acute cellular rejection	13 (14.6%)	6 (10.2%)	0.713
Antibody-mediated rejection	1 (1.1%)	1 (1.7%)	0.768
Graft failure within 90 days	0 (0%)	2 (3.4%)	0.157
Death within 90 days	0 (0%)	3 (5.1%)	0.061
Follow-up duration (months)	44.5 ± 17.6	38.8 ± 19.2	0.110

*GRWR, graft to recipient weight ratio.

**Figure 1:**
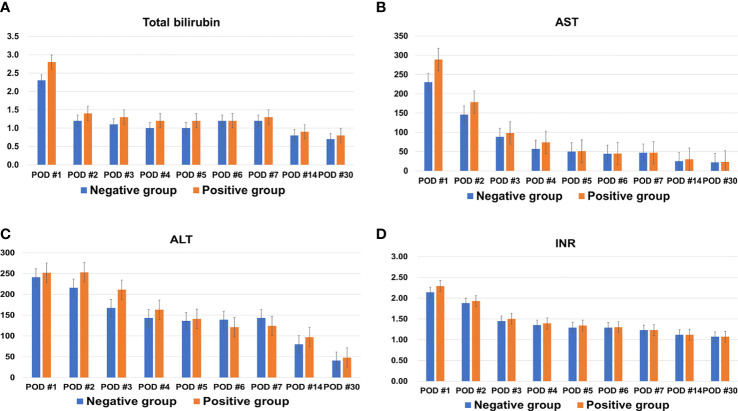
Changes in **(A)** total bilirubin, **(B)** aspartate transaminase (AST), **(C)** alanine transaminase (ALT), and **(D)** international normalized ratio (INR) of both groups within one month after living donor liver transplantation.

Median hospitalization time was longer in the positive group than the negative group (27 days vs. 23 days; P=0.048). Total bilirubin, AST, ALT, and INR, tested repeatedly up to 30 days after LDLT, did not differ between the two groups (**Figure 1**). None of the following measures were significantly different between the two groups: ABO-incompatibility, cold and warm ischemic times, recipient operation time, early allograft dysfunction, intensive care unit stay, incidence of values beyond Milan criteria for tumor pathology, tumor size, tumor number, presence of total tumor necrosis, tumor grade 3 or 4, encapsulation, presence of portal vein tumor thrombosis (PVTT), intrahepatic metastasis, and multicentric occurrence.

The incidences of bacterial, viral, and surgical complications within 90 days after LDLT were not different between the two groups. Accordingly, the severity of Clavien-Dingo grade was not different between the two groups.

### Outcomes

Mean follow-up duration was 44.5 ± 17.6 months in the negative group and 38.8 ± 19.2 months in the positive group. The incidence of acute cellular rejection across the follow-up period was 14.6% (n=13) in the negative group and 10.2% (n=6) in the positive group. Only one patient in each group had antibody-mediated rejection.

**Figure 2:**
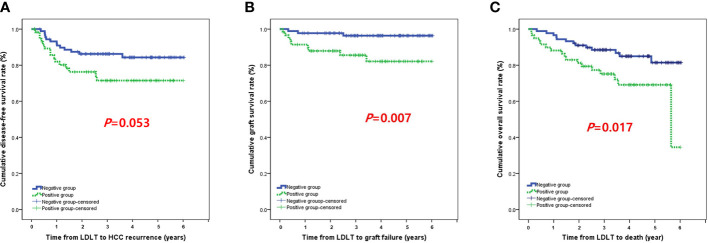
**(A)** Disease-free survival, **(B)** death-censored graft survival, and **(C)** patient survival.

The incidence of HCC recurrence was 14.6% (n=13) in the negative group and 25.4% (n=15) in the positive group. The 1-, 2-, and 3-year cumulative disease-free survival rates were 92.0%, 86.2%, and 86.2%, respectively, in the negative group and 82.0%, 76.3%, and 71.5% in the positive group (P=0.053; **Figure 2A**). Three patients (3.4%) in the negative group and nine patients (15.3%) in the positive group developed graft failure during the follow-up period. The 1-, 2-, and 3-year cumulative death-censored graft failure rates were 97.8%, 97.8%, and 96.4%, respectively, in the negative group and 91.4%, 85.5%, and 82.1% in the positive group (P=0.007; **Figure 2B**). The incidence of death was 14.6% (n=13) in the negative group and 28.8% (n=17) in the positive group. The 1-, 2-, and 3-year cumulative overall survival rates were 96.6%, 91.0%, and 88.5%, respectively, in the negative group and 88.1%, 81.2%, and 75.1% in the positive group (P=0.017; **Figure 2C**).

### Risk Factors for Hepatocellular Carcinoma Recurrence, Death-Censored Graft Failure, and Death

Risk factors for HCC recurrence, death-censored graft failure, and death are summarized in **Supplement Tables 1**, **2**, and **3**. Multivariate analysis showed that young recipient age, the positive group, being beyond the Milan criteria, and intrahepatic metastasis were predisposing factors for HCC recurrence. Death-censored graft failure and death were strongly associated with the positive group (**Table 3**).

**Table 3:** Risk factors in multivariate analysis.

	HR	95% CI	P-value
**HCC recurrence**			
Recipient age	0.925	0.859–0.997	0.041
Graft weight change (Positive group)	2.883	1.280–6.492	0.011
Beyond Milan criteria	3.470	1.030–11.696	0.045
Intrahepatic metastasis	5.158	1.506–17.669	0.009
**Death-censored graft failure**			
Graft weight change (Positive group)	5.280	1.395–19.985	0.014
**Death**			
Graft weight change (Positive group)	3.751	1.628–8.642	0.002

*HR, hazard ratio; 95% CI, 95% confidence interval; HCC, hepatocellular carcinoma.

## Discussion

Because graft quality affects recipient’ outcome, numerous studies have investigated the effects of graft volume, fatty changes in donor liver, and GRWR on recipient outcome after LDLT (2). However, no previous study has investigated the effect of change in graft weight after perfusion on LDLT outcome.

In previous studies, Graft weight measurement timing is not mentioned or measured at various times and measured immediately after procurement, or after preservation solution, or after back-table procedure (8–10). Previous studies showed that careful interpretation of liver graft weight is required because graft weight after back-bench surgery can decrease to 90% of the initial graft weight when using University of Wisconsin (UW) solution for perfusion because the solution is lighter than blood. However, LDLT in our study used HTK solution, and one-third of patients experienced liver graft weight increases after procurement, which is inconsistent with previous findings.

Passive volume changes according to HTK solution perfusion would be expected to be quite small. Individual liver graft compliance was clinically observed as the discrepancy between perfused liver graft weight and non-perfused liver graft weight at the time of retrieval. In this study, median graft weight decreased by 28 g in the negative group and increased 21 g in the positive group. Weight change was very small because the liver has a large capacitance reservoir and very low venous resistance.

This study showed no significant difference in donor characteristics between the two groups. On the recipient side, hepatitis C virus (HCV) and alcoholic patients in the positive group were slightly more prevalent in the negative group, and median hospitalization in the positive group was longer than that in the negative group. There were no statistically significant differences in tumor characteristics, postoperative complications, EAD, or acute rejection between the two groups. Interestingly, disease-free survival, death-censored graft survival, and patient survival were lower in the positive group than the negative group. Additionally, the positive group was strongly associated with HCC recurrence, death-censored graft survival, and patient survival in multivariate analysis.

We suspect that the increase in liver graft weight after HTK solution perfusion was due to deficiency in liver graft elasticity, which is thought to have adverse effects the patient after graft implantation. The increase in kidney graft weight during perfusion showed vascular disruption by endothelial disruption and inflammatory infiltration (11). The volume HTK solution in the liver vascular bed has not been well examined in our study. The increase in liver graft weight after HTK solution perfusion that could reflect hepatocyte parenchymal edema. Liver graft edema may destroy the endothelial surface layer along with the inevitable damage caused by surgical trauma (such as mechanical stress and ischemia-reperfusion injury), leading to pathologic shifts of fluid and protein towards the interstitium (12). Disruption of electrolyte cell membrane gradients due to sodium-potassium membrane pumps damage results in cellular edema, with free calcium influx, and subsequent activation of enzyme cascade leading to cell death (13). Furthermore, pathologic inflammation and endothelial dysfunction are known to promote angiogenesis, fibrogenesis, cirrhosis, and increased hepatic resistance, ultimately resulting in portal hypertension and decreased effective hepatocyte perfusion with the risk of liver failure (14).

After graft implantation, hepatocyte injury after reperfusion is mediated by release of reactive oxygen species with subsequent oxidative stress (8). In addition, when Kupffer cells and sinusoidal endothelial cells in the partial liver graft are exposed to high portal venous blood flow, shear stress caused by high portal venous blood flow induces hepatic regeneration (15). Decreased liver graft elasticity might affect early outcomes after LDLT. Therefore, we tested liver function tests and INR repeatedly until one month after LDLT to compare levels between the two groups because the most appropriate time to evaluate graft regeneration in the early postoperative period is during the second postoperative week (15, 16). However, there was no difference in aspartate transaminase (AST), alanine transaminase (ALT), total bilirubin, or INR between the two groups. Additionally, there were no significant differences in EAD, acute rejection, and postoperative surgical complications between the two groups. Positive liver graft weight change does not seem to have a significant early effect after LDLT.

HCC recurrence after LDLT increases the likelihood of death in liver transplant patients. The most common risk factors for HCC recurrence after liver transplantation were tumor size and tumors, tumor differentiation, tumor markers included AFP or PIVKA-II, vascular invasion, and neutrophil-lymphocyte ratio (17). The AFP model was a well-validated preoperative risk model for stratifying patients into high- and low-risk groups (18).

HCC patients may have a higher probability of HCC recurrence after LDLT than DDLT. When partial liver graft is implanted, growth factor and vascular inflow increase for regeneration of liver graft (19). This process may contribute to progression and seeding in any organs of tumor cells in blood. Additionally, small size grafts are more likely to induce HCC recurrence due to cell adhesion, angiogenesis, and migration caused by acute phase graft injury and rapid graft regeneration (20). Hepatic fibrosis reflects liver function and shows the possibility of cirrhosis and HCC development in patients with chronic liver disease (21). Elastography-based imaging techniques have emerged as an accurate method for assessing liver fibrosis. High liver stiffness and transient elastography results were significantly correlated with HCC occurrence in chronic liver disease patients without a history of HCC (22). An increase in liver graft weight is likely to be related to liver graft elasticity, which may have a synergistic effect on HCC recurrence through the various factors described above that affect HCC recurrence in LDLT. Our study showed that the positive group had a higher likelihood of death-censored graft survival and patient survival in multivariate analysis; therefore, liver graft elasticity may eventually affect graft survival and patient survival.

This study has several imitations. First, we do not know the degree of liver stiffness of the liver donor, so it is difficult to measure liver elasticity precisely. Thus, we do not know the relationship between liver graft weight change and liver elasticity. Second, we were not able to control for confounding factors because of the retrospective nature of this observational study. In addition, it is difficult to completely remove blood and HTK solution in the graft and exactly measure the liver graft weight. The study population was small and enrolled from a single center, making it difficult to obtain high statistical power. Third, biological factors before and after perfusion were unknown in present study. Prospective research on peripheral blood, perfusate solutions, and liver biopsy before and after perfusion is required. Fourth, we are not sure how positive liver graft weights change contribute to HCC recurrence, graft survival, or patient death. Randomized controlled prospective studies are required to validate our findings.

This study showed that positive graft weight change during HTK solution perfusion strongly contributed to HCC recurrence, graft failure, and patient death. However, this change in graft weight change has no effect initially after LDLT. Our study revealed that positive graft weight change during HTK solution perfusion is an indicator of poor prognosis in LDLT patients. However, we do not know the exact mechanism between graft weight change and poor prognosis. More research is needed to clarify this mechanism or relationship.

## Data Availability Statement

The datasets generated for this study are available on request to the corresponding author.

## Ethics Statement

This study was approved by the Samsung Medical Center Institutional Review Board (IRB) (SMC-2020-07-027). Patient consent was waived by the IRB. Written informed consent was not obtained from the individual(s) for the publication of any potentially identifiable images or data included in this article.

## Author Contributions

JMK designed the study, conducted the literature search, acquired, analyzed, and interpreted the data and wrote the manuscript. J-WJ and YJC designed the study and interpreted the data. SK analyzed the data. JR and G-SC acquired and analyzed the data. All authors read and approved the final manuscript. All authors contributed to the article and approved the submitted version.

## Conflict of Interest

The authors declare that the research was conducted in the absence of any commercial or financial relationships that could be construed as a potential conflict of interest.

## Abbreviations

HCC, hepatocellular carcinoma; MELD, model for end-stage liver disease; GRWR, graft-to-recipient ratio; HTK, Histidine-Tryptophan-Ketoglutarate; EAD, early allograft dysfunction; DFS, disease-free survival; PS, patient survival; BMI, body mass index; HBV, hepatitis B virus; AFP, alpha-fetoprotein; PIVKA-II, protein induced by vitamin K absence-II; HCV, hepatitis C virus; AST, aspartate transaminase; ALT, alanine transaminase.
