# Supplementary material for: Impact of Graft Weight Change During Perfusion on Hepatocellular Carcinoma Recurrence After Living Donor Liver Transplantation
Source: Front Oncol. 2021 Feb 24;10:609844. doi: 10.3389/fonc.2020.609844 (PMC7945034; doi:10.3389/fonc.2020.609844)
Supplement: Supplementary file 1 [file DataSheet_1.docx]

Table 1. Risk factors for HCC recurrence in univariate analysis

|  | HR | 95% CI | P-value |
| --- | --- | --- | --- |
| Gender (Male) | 1.331 | 0.316-5.609 | 0.697 |
| Age | 0.925 | 0.867-0.988 | 0.020 |
| ABO-incompatibility | 1.174 | 0.517-2.666 | 0.701 |
| Laparoscopic donor hepatectomy | 0.808 | 0.356-1.834 | 0.610 |
| Early allograft dysfunction | 1.401 | 0.190-10.318 | 0.741 |
| MELD | 1.019 | 0.958-1.083 | 0.556 |
| Graft weight change (Positive group) | 2.047 | 0.973-4.304 | 0.059 |
| GRWR after perfusion | 1.421 | 0.242-8.344 | 0.697 |
| Macrosteatosis | 0.958 | 0.864-1.062 | 0.414 |
| Microsteatosis | 0.925 | 0.845-1.013 | 0.094 |
| Operation time in living donor | 1.003 | 0.998-1.008 | 0.203 |
| Operation time in recipient | 1.002 | 0.998-1.005 | 0.309 |
| Cold ischemic time | 0.998 | 0.983-1.014 | 0.847 |
| Warm ischemic time | 1.014 | 0.988-1.041 | 0.301 |
| Recipient body mass index | 0.949 | 0.850-1.059 | 0.347 |
| Child-Pugh class  A  B  C | 1  1.401  1.216 | 1  0.595-3.300  0.450-3.289 | 0.741  0.441  0.700 |
| Hypertension | 0.040 | 0.000-4.767 | 0.187 |
| Diabetes | 0.746 | 0.258-2.158 | 0.589 |
| Intensive care unit stay | 0.988 | 0.809-1.207 | 0.907 |
| Hospitalization | 1.006 | 1.001-1.010 | 0.023 |
| Acute cellular rejection | 0.804 | 0.241-2.678 | 0.722 |
| History of hepatectomy | 2.087 | 0.882-4.938 | 0.094 |
| History of radiation | 1.596 | 0.480-5.308 | 0.445 |
| Donor sex (Male) | 1.096 | 0.492-2.440 | 0.823 |
| Donor age | 0.993 | 0.961-1.026 | 0.661 |
| Donor body mass index | 0.990 | 0.870-1.127 | 0.880 |
| AFP ≥10 | 1.127 | 0.523-2.430 | 0.760 |
| PIVKA-II ≥40 | 2.560 | 1.198-5.471 | 0.015 |
| Beyond Milan criteria | 3.266 | 1.513-7.050 | 0.003 |
| Tumor size | 1.232 | 0.988-1.536 | 0.063 |
| Tumor number | 1.021 | 0.933-1.117 | 0.651 |
| Total tumor necrosis | 1.349 | 0.631-2.884 | 0.440 |
| Tumor grade 3 or 4 | 0.755 | 0.227-2.509 | 0.647 |
| Encapsulation | 1.239 | 0.500-3.067 | 0.643 |
| Microvascular invasion | 2.335 | 1.083-5.038 | 0.031 |
| Portal vein tumor thrombosis | 1.851 | 0.438-7.831 | 0.403 |
| Intrahepatic metastasis | 3.014 | 1.406-6.459 | 0.005 |
| Multicentric occurrence | 1.123 | 0.474-2.656 | 0.792 |

* HR, hazard ratio; MELD, Model for End-Stage Liver Disease; GRWR, graft to recipient weight ratio; AFP, alpha-fetoprotein; PIVKA-II, proteins induced by vitamin K absence or antagonist-II

Table 2. Risk factors for death-censored graft survival in univariate analysis

|  | HR | 95% CI | P-value |
| --- | --- | --- | --- |
| Gender (Male) | 1.130 | 0.146-8.756 | 0.907 |
| Age | 1.058 | 0.963-1.161 | 0.240 |
| ABO-incompatibility | 2.186 | 0.694-6.889 | 0.182 |
| Early allograft dysfunction | 0.048 | 0.000-40.186 | 0.709 |
| MELD | 1.053 | 0.974-1.139 | 0.195 |
| Graft weight change (Positive group) | 5.071 | 1.371-18.753 | 0.015 |
| GRWR after perfusion | 8.609 | 0.831-90.206 | 0.071 |
| Macrosteatosis | 1.085 | 0.973-1.210 | 0.142 |
| Microsteatosis | 0.969 | 0.873-1.074 | 0.547 |
| Operation time in living donor | 1.005 | 0.998-1.012 | 0.176 |
| Operation time in recipient | 1.003 | 0.998-1.007 | 0.255 |
| Cold ischemic time | 1.008 | 0.984-1.032 | 0.506 |
| Warm ischemic time | 0.953 | 0.905-1.004 | 0.068 |
| Recipient body mass index | 0.960 | 0.810-1.138 | 0.639 |
| Varix bleeding | 0.043 | 0.000-49.190 | 0.510 |
| Ascites |  |  |  |
| Child-Pugh class  A  B  C | 1  1.949  1.086 | 1  0.523-7.263  0.199-5.930 | 0.570  0.320  0.924 |
| Hypertension | 1.643 | 0.355-7.606 | 0.525 |
| Diabetes | 1.581 | 0.419-5.963 | 0.499 |
| Intensive care unit stay | 1.170 | 0.944-1.449 | 0.151 |
| Hospitalization | 1.002 | 0.991-1.012 | 0.756 |
| Acute cellular rejection | 2.266 | 0.601-8.546 | 0.227 |
| History of hepatectomy | 3.027 | 0.886-10.344 | 0.077 |
| History of radiation | 4.023 | 1.066-15.174 | 0.040 |
| Donor sex (male) | 0.961 | 0.281-3.286 | 0.950 |
| Donor age | 1.004 | 0.956-1.055 | 0.862 |
| Donor body mass index | 0.913 | 0.731-1.142 | 0.426 |
| AFP ≥10 | 1.977 | 0.603-6.482 | 0.261 |
| PIVKA-II ≥40 | 3.307 | 0.968-11.299 | 0.056 |
| Beyond Milan criteria | 1.744 | 0.532-5.717 | 0.359 |

* HR, hazard ratio; MELD, Model for End-Stage Liver Disease; GRWR, graft to recipient weight ratio; AFP, alpha-fetoprotein; PIVKA-II, proteins induced by vitamin K absence or antagonist-II

Table 3. Risk factors for death in univariate analysis

|  | HR | 95% CI | P-value |
| --- | --- | --- | --- |
| Gender (Male) | 0.850 | 0.257-2.811 | 0.790 |
| Age | 0.984 | 0.929-1.042 | 0.579 |
| ABO-incompatibility | 1.285 | 0.588-2.810 | 0.529 |
| Laparoscopic donor hepatectomy | 1.157 | 0.536-2.494 | 0.711 |
| Early allograft dysfunction | 1.322 | 0.180-9.728 | 0.784 |
| MELD | 1.019 | 0.963-1.079 | 0.506 |
| Graft weight change (Positive group) | 2.355 | 1.140-4.866 | 0.021 |
| GRWR after perfusion | 6.899 | 1.462-32.563 | 0.015 |
| Macrosteatosis | 1.058 | 0.983-1.139 | 0.133 |
| Microsteatosis | 0.963 | 0.899-1.030 | 0.273 |
| Operation time in living donor | 1.003 | 0.998-1.008 | 0.295 |
| Operation time in recipient | 1.002 | 0.999-1.005 | 0.249 |
| Cold ischemic time | 1.000 | 0.984-1.015 | 0.984 |
| Warm ischemic time | 1.000 | 0.974-1.027 | 0.971 |
| Recipient body mass index | 0.970 | 0.872-1.080 | 0.582 |
| Varix bleeding | 1.226 | 0.369-4.073 | 0.740 |
| Ascites |  |  |  |
| Child-Pugh class  A  B  C | 1  1.767  1.273 | 1  0.759-4.113  0.470-3.448 | 0.416  0.187  0.635 |
| Hypertension | 0.542 | 0.129-2.286 | 0.404 |
| Diabetes | 0.940 | 0.356-2.483 | 0.900 |
| Intensive care unit stay | 1.043 | 0.861-1.263 | 0.670 |
| Hospitalization | 1.001 | 0.994-1.009 | 0.750 |
| Acute cellular rejection | 1.070 | 0.370-3.097 | 0.900 |
| History of hepatectomy | 2.070 | 0.911-4.705 | 0.082 |
| History of radiation | 1.381 | 0.416-4.580 | 0.598 |
| Donor sex (male) | 0.781 | 0.363-1.679 | 0.526 |
| Donor age | 1.013 | 0.983-1.043 | 0.410 |
| Donor body mass index | 1.015 | 0.894-1.153 | 0.813 |
| AFP ≥10 | 1.606 | 0.765-3.371 | 0.210 |
| PIVKA-II ≥40 | 1.623 | 0.722-3.413 | 0.202 |
| Beyond Milan criteria | 1.779 | 0.844-3.750 | 0.130 |
| Tumor size | 1.259 | 0.988-1.603 | 0.063 |
| Tumor number | 1.087 | 1.030-1.148 | 0.002 |
| Total tumor necrosis | 2.106 | 0.994-4.461 | 0.052 |
| Tumor grade 3 or 4 | 0.457 | 0.108-1.926 | 0.286 |
| Encapsulation | 1.148 | 0.505-2.610 | 0.741 |
| Microvascular invasion | 1.560 | 0.743-3.276 | 0.240 |
| Portal vein tumor thrombosis | 0.845 | 0.114-6.237 | 0.869 |
| Intrahepatic metastasis | 1.927 | 0.888-4.180 | 0.097 |
| Multicentric occurrence | 1.541 | 0.697-3.410 | 0.286 |

* HR, hazard ratio; MELD, Model for End-Stage Liver Disease; GRWR, graft to recipient weight ratio; AFP, alpha-fetoprotein; PIVKA-II, proteins induced by vitamin K absence or antagonist-II
